# Effects of yeast, tannin, and amylolytic enzyme combinations in high-concentrate diets on feed intake, nutrient digestibility, and serum metabolites in Lambs

**DOI:** 10.1007/s11250-026-05299-w

**Published:** 2026-09-09

**Authors:** Lucas Eduardo Gonçalves Vilaça, Erica Beatriz Schultz, Marcela Rodrigues de Oliveira, Karla Alves Oliveira, Marco Túlio Santos Siqueira, Pedro Henrique Cavalcante Ribeiro, Gilberto de Lima Macedo Júnior

**Affiliations:** 1 Departamento de Zootecnia, Universidade Federal de Viçosa, Minas Gerais, Brasil; 2 Departamento de Medicina Veterinária e Zootecnia, Universidade Federal de Uberlândia, Minas Gerais, Brasil; 3 EMBRAPII, Instituto Federal do Triangulo Mineiro, Uberaba, Minas Gerais Brasil; 4 Departamento de Zootecnia, Universidade Estadual Paulista, São Paulo, Brasil

**Keywords:** Amylolytic enzymes, High-concentrate diet, Lambs, Saccharomyces cerevisiae, Tannins, Nutrient digestibility

## Abstract

The purpose of this study was to evaluate the combined effects of yeast, tannin, and amylolytic enzymes on intake, digestibility, and biochemical parameters of lambs fed a high-concentrate diet. A total of five lambs were used in a 5 × 5 Latin square design. Diets consisted of 75% concentrate and 25% roughage. The treatments were: control (without additive), TE (tannin and enzyme), EY (enzyme and yeast), TY (tannin and yeast), and TYE (tannin, yeast, and enzyme). Digestibility trials were conducted to determine nutrient intake and digestibility, as well as urinary, fecal, and serum parameters. Analyses of variance were applied, and means were compared using Tukey’s test at a 5% significance level. No differences were observed in nutrient intake or urinary and fecal parameters with the inclusion of feed additives (*P* > 0.05). However, dry matter digestibility increased with additive inclusion (775 vs. 749 g kg^− 1^ day^− 1^, *P* = 0.04). Crude protein digestibility decreased (886 vs. 912 g kg^− 1^ day^− 1^, *P* < 0.0096) with the combination of tannin and yeast. For metabolites, serum concentrations of gamma-glutamyl transferase (28.5 vs. 25 U/L, *P* = 0.01) increased, whereas alkaline phosphatase (272 vs. 331 U/L, *P* = 0.02) decreased with the use of dietary additive. The results suggest that the inclusion of yeast, tannin, and amylolytic enzymes in high-concentrate diets for lambs may contribute to increased dry matter digestibility and changes in selected hepatic parameters, without compromising feed intake or animal health.

## Introduction

Ruminant production systems have intensified, leading to the adoption of diets with higher proportions of rapidly fermentable carbohydrates to improve growth performance, mainly during the finishing phase in feedlot (Zhang et al. 2022). Nevertheless, increasing concentrate levels may compromise ruminal fermentation and metabolic health. The use of feed additives represents an alternative strategy to modulate the ruminal environment, improve nutrient utilization, and mitigate metabolic disorders associated with high-concentrate diets (Song et al. 2021).

Among feed additives, yeasts, tannins, and enzymes draw attention for their functional roles in improving ruminal efficiency and animal health. Yeasts are feed additives composed of live microorganisms that, when incorporated into the diet, can modulate the ruminal and intestinal microbial ecosystem (Uyeno et al. 2015). As an example, *Saccharomyces cerevisiae* has been shown to stabilize ruminal pH, stimulate microbial growth, and enhance fiber digestibility (Song et al. 2021). Tannins are a heterogeneous group of polyphenolic compounds capable of binding to proteins and carbohydrates, forming stable complexes that decrease the ruminal degradation of nutrients, thereby enhancing nitrogen utilization efficiency (Besharati et al. 2022). Exogenous enzymes, particularly amylolytic enzymes, serve as catalysts that promote dietary starch degradation in high-concentrate diets, improving energy availability and reducing the incidence of metabolic disorders (Ncube et al. 2014).

Song et al. (2021), Azzaz et al. (2020), and Besharati et al. (2022) reported that combinations of feed additives can improve digestive and metabolic parameters, although their effects are dose dependent. While tannins may reduce protein digestibility in some cases, they have also been associated with improved nitrogen utilization efficiency and reduced urea excretion (Besharati et al. 2022). Similarly, amylolytic enzymes may counteract the negative effects of tannins on starch digestion when used in combination (Azzaz et al. 2020).

In sheep diets, Siqueira et al. (2022) observed that the inclusion of active and inactive yeasts combined with fibrolytic enzymes did not affect dry matter intake or digestibility in ewes but improved protein digestibility. On the other hand, Rodrigues et al. (2022) showed that different combinations of exogenous enzymes increased nutrient intake and digestibility, as well as serum urea concentrations. Neiva et al. (2022) found increased nutrient intake accompanied by a higher nitrogen balance (NB). Wang et al. (2023) observed that supplementing 0.5% tannin in sheep diets significantly reduced serum triglyceride and total cholesterol concentrations, possibly due to modulation of the intestinal microbiota. These findings highlight the existing inconsistencies and the need for further elucidation regarding the combined use of feed additives in ruminant diets.

Despite the reported effects of individual and combined feed additives, there is still a lack of consistent evidence regarding their interactive effects, particularly under high-concentrate feeding conditions, which are known to challenge ruminal and metabolic homeostasis. In addition, most studies have evaluated these additives in isolation, and the potential synergistic or antagonistic interactions among yeast, tannins, and amylolytic enzymes remain poorly understood (Azzaz et al. 2020; Besharati et al. 2022; Rodrigues et al. 2022; Song et al. 2021; Wang et al. 2023). This gap limits the ability to establish effective nutritional strategies for optimizing performance and metabolic health in intensive production systems.

Therefore, our hypothesis was that the combination of feed additives would not affect nutrient intake but would enhance digestibility and modulate metabolic responses in lambs fed high-concentrate diets. Accordingly, this study aimed to evaluate the effects of combining live yeast, tannins, and amylolytic enzymes on nutrient intake and digestibility, serum metabolites, urinary, and fecal parameters of lambs.

## Materials and methods

The experiment was conducted at the Federal University of Uberlândia (UFU), Uberlândia, Minas Gerais, Brazil, from October 23, 2023, to January 5, 2024, totaling 75 days.

### Animals and experimental design

Five crossbred female lambs (Dorper × Santa Inês), with an average age of 4 ± 0.8 months and an initial body weight of 16.75 ± 4.62 kg were distributed in a 5 × 5 Latin square design. Lambs were housed in individual metabolic cages of 2 m^2^ provided with feeders and drinking fountains. The total experimental period was 75 days, divided into five phases, each consisting of the first ten days for adaptation and five days for data collection.

During each collection period, daily records were taken for feed offered, leftovers, total fecal output, water intake, fecal score, and urinary volume and density. Animals were weighed at the beginning and end of each period using a Kapbom^®^ digital scale (Kapbom Indústria e Comércio Ltda., Cravinhos, SP, Brazil; accuracy: 50 g) after a 16-hour fast, to determine body weight variation and adjust feed intake.

### Treatments

The experimental diets were formulated according to NRC (2007). The forage-to-concentrate ratio was 25:75. The treatments were: control (diet without additive), TE (tannin and enzyme), EY (enzyme and yeast), TY (tannin and yeast), and TYE (tannin, yeast and enzyme). Additives were weighed daily using a Shimadzu^®^ analytical balance (Shimadzu Corporation, Kyoto, Japan) with a precision of 0.001 g and mixed with the offered diet. Dosages were adjusted based on the animals’ dry matter intake (DMI) and consisted of 0.5 g of amylolytic enzyme (Amaize^®^, Alltech Inc., Nicholasville, KY, USA) per kg of DM; 0.15 g of tannin (Silvafeed^®^ BX, containing 62% tannins from a mixture of chestnut and quebracho; Silvateam, San Michele Mondovì, Italy) per kg of DM; and 2 g of live yeast (Yea-Sacc^®^, Saccharomyces cerevisiae, 5 × 10⁹ CFU/g; Alltech Inc., Nicholasville, KY, USA) per kg of DM (Table 1).

**Table 1:** Ingredients and chemical composition of the diet

Ingredients			kg / 100 kg			
Ground corn			71.0			
Soybean meal			23.0			
Urea			1.0			
Mineral Mixture			5.0			
Additive			kg/ DMI			
Yeast¹			2.00 g			
Tannin²			0.15 g			
Amylolytic Enzyme³			0.50 g			
Chemical Composition		Item				
		Concentrate	Corn Silage	Total Diet		
Dry Matter (%)		91.84	33.16	77.17		
Crude Protein (%)		27.76	9.56	22.64		
Neutral Detergent Fiber (%)		18.22	59.18	28.45		
Starch (%)		73.00	30.00	62.25		
Ash (%)		6.26	4.58	10.52		

¹Live yeast Yea-Sacc^®^ (*Saccharomyces cerevisiae*, 5 × 10⁹ CFU/g), Alltech^®^ (Alltech Inc., Nicholasville, USA); ²Silvafeed^®^ BX tannin (Silvateam S.p.A., San Michele Mondovì, Italy), composed of a mixture of chestnut and quebracho tannins (62%); ³Amylolytic enzyme Amaize^®^ Alltech^®^ (Alltech Inc., Nicholasville, USA); kg/DMI: kilograms per kilogram of dry matter intake

These inclusion levels were established according to the manufacturers’ recommendations, which differ among additives due to variations in their active compounds, biological activity, and intended mode of action. The selected doses were designed to ensure efficacy while maintaining dietary safety and practical applicability under production conditions.

### Feeding management

Animals were fed ad libitum twice daily, at 08:00 and 16:00 h. Diets were weighed prior to feeding using a Junipi^®^ electronic scale (Junipi Balanças Ltda., São Paulo, SP, Brazil) with an accuracy of 5 g. Feed leftovers were measured daily in the morning and adjusted to maintain approximately 10% of the feed offered as leftovers. Feed intake was determined by the difference between the amount of feed offered and the leftovers recorded.

Daily samples of the feed offered and leftovers from each animal were collected to obtain a composite sample representing the five-day digestibility period of each experimental phase. Samples were placed in labeled plastic bags and stored in a freezer at − 15 °C for subsequent chemical analyses.

Water intake was determined daily as the difference between the amount offered and the leftovers, using 8 L containers per animal, with corrections for water evaporation. To estimate water evaporation over a 24-hour period, an 8 L container filled with water was placed daily in the experimental barn, in a location inaccessible to the animals and at the same height as the buckets in the metabolic cages. Evaporation was quantified as the difference between the 8 L of water offered and the amount remaining the following morning. Measurements were performed using 2 L graduated plastic cylinders (Jprolab^®^, Jprolab Equipamentos Científicos Ltda., São Paulo, SP, Brazil) with an accuracy of 20 mL.

### Collection and analysis of feces and urine

Total urine collection was performed using containers with 100 mL of sulfuric acid (H₂SO₄) to prevent nitrogen (N) volatilization and microbial fermentation. Screens were placed above the containers to retain feces and avoid contamination. Urine was collected daily in the morning, and its volume was measured using 2 L graduated plastic cylinders (Jprolab^®^, Jprolab Equipamentos Científicos Ltda., São Paulo, SP, Brazil; accuracy: 20 mL).

For each animal, 20% of the daily urine volume was sampled into 2 L plastic containers throughout the five-day collection period to obtain a composite sample. Before subsampling, the urine was thoroughly homogenized to ensure representativeness. Samples were filtered, transferred to labeled 200 mL plastic bottles, and stored at − 15 °C for nitrogen analysis.

Urinary nitrogen content was determined using the Kjeldahl method (Silva and Queiroz, 2002) with minor adaptations. Briefly, 1 mL of urine, 5 mL of sulfuric acid, and a catalytic mixture were digested with a gradual temperature increase up to color change. The digested sample was then distilled with 25 mL of 50% sodium hydroxide (NaOH) and 20 mL of boric acid (H₃BO₃), and the distillate was titrated with 0.1 N hydrochloric acid (HCl) to determine total nitrogen concentration.

Fecal consistency was visually evaluated using a five-point scoring system, as described by Dickson and Jolly (2011): 1 – dry, dull feces; 2 – normal consistency; 3 – soft feces, partially losing shape and sticking together (grape-like appearance); 4 – soft, shapeless feces (similar to pig feces); and 5 – diarrheic feces. Scoring was performed during the five-day collection period by a single evaluator to minimize variation.

Feces were weighed and sampled daily in portions of approximately 100 g in the morning, totaling 500 g of sample per animal at the end of each collection period. After collection, samples were homogenized and stored at − 15 °C for subsequent chemical analyses.

### Chemical composition analyses

Samples of feed offered, leftovers, and feces were analyzed for chemical composition according to Detmann et al. (2021) to determine dry matter (DM), crude protein (CP), ash (ASH), neutral detergent fiber (NDF), acid detergent fiber (ADF), and starch contents. After determining the chemical composition, nutrient intake and digestibility were calculated.

### Blood sampling and biochemical analyses

Blood samples (4 mL per collection) were collected from the jugular vein on days 11, 13, and 15 of each experimental period, corresponding to the collection phase following the dietary adaptation period. These sampling days were selected to evaluate serum biochemical parameters after the animals had been exposed to the experimental diets for a sufficient period to achieve metabolic stabilization and to monitor possible temporal variations in the measured variables. Blood was collected using Vacutainer^®^ tubes containing a clot activator (silica) (BD, São Paulo, SP, Brazil). All collections were performed at 08:00 h with animals fasted. Samples were centrifuged at 3,500 rpm for 15 min, and the serum was separated, aliquoted into labeled 1.5 mL Eppendorf tubes, and stored at − 20 °C until analysis.

Serum concentrations of fructosamine, cholesterol, triglycerides, high-density lipoprotein (HDL), low-density lipoprotein (LDL), gamma-glutamyl transferase (GGT), alkaline phosphatase (ALP), aspartate aminotransferase (AST), albumin, total protein, urea, uric acid, and creatinine were determined using a PKL 125^®^ automatic biochemical analyzer (PKL Instrumentos Ltda., São Paulo, SP, Brazil) with commercial reagents from Labtest^®^ (Labtest Diagnóstica S.A., Lagoa Santa, MG, Brazil). Results were expressed as the mean of the three samples collected during each experimental period.

### Statistical analysis

The experiment was conducted as a 5 × 5 Latin square design, consisting of five animals, five treatments, and five experimental periods. This design allowed each animal to receive all treatments across periods while controlling for animal and period effects, thereby increasing the precision of treatment comparisons and reducing experimental variability. The number of animals used was determined by the structure of the Latin square design, in which the number of animals, treatments, and periods must be equal.

Data were analyzed according to the following mathematical model: $\:{\mathrm{Y}}_{\mathrm{i}\mathrm{j}\mathrm{k}}=\:{\upmu\:}+\:{\mathrm{T}}_{\mathrm{i}}+\:{\mathrm{P}}_{\mathrm{j}}+\:{\mathrm{A}}_{\mathrm{k}}+\:{\mathrm{e}}_{\mathrm{i}\mathrm{j}\mathrm{k}}$; where µ is the overall mean, $\:{T}_{i\:}$is the fixed effect of treatment, $\:{P}_{j}\:$is the random effect of period, $\:{A}_{k\:}$is the random effect of animal, and $\:{e}_{ijk}\:$is the residual error.

The assumptions of normality and homogeneity of residual variances were verified prior to analysis. Data were analyzed using R software (R Core Team, 2023). The treatment effect was evaluated using the F-test, and means were compared using Tukey’s test. In addition to the overall treatment comparison, a planned contrast analysis was performed. The Control vs. Additive contrast compared the control treatment (without additives) with the average response of all additive-containing treatments (TE, EY, TY, and TYE). The fecal score data were analyzed using non-parametric statistical methods due to the ordinal nature of the variable. Initially, differences among treatments were evaluated using the Kruskal–Wallis test. When significant effects were detected, pairwise comparisons were performed using the Wilcoxon test to identify differences between treatments. Statistical significance was declared at *P* ≤ 0.05.

## Results

### Feed intake, digestibility, and nitrogen balance

The inclusion of feed additives in lamb diets did not affect dry matter intake (DMI), expressed either as g/day or % BW, nor starch intake (SI), crude protein intake (CPI), neutral detergent fiber intake (NDFI), or neutral detergent fiber digestibility (DNDF) (*P* > 0.05; Table 2). These results indicate that additive supplementation did not alter nutrient consumption under the conditions of the present study.

**Table 2:** Nutrient intake and digestibility in lambs fed diets with yeast, tannins, amylolytic enzymes, or without additive

Item	Control	EY	TE	TY	TYE	SEM	*p*-value	Control vs. Additive
DMI, kg day^− 1^	1.15	1.40	1.31	1.35	1.33	0.04327	0.3573	0.1481
DMI, % BW	5.39	5.66	5.65	6.30	5.97	0.1749	0.1752	0.1031
DMI, g/MBW	116	123	125	136	131	3.4516	0.1960	0.0672
SI, g day^− 1^	845	922	882	853	790	35.2	0.7625	0.8842
CPI, g day^− 1^	250	262	307	231	278	10.9	0.7892	0.9546
NDFI, g day^− 1^	312	313	370	314	345	15.03	0.6659	0.6315
DDM, g/Kg DM	749	785	764	768	786	4.93	0.1346	0.0499
DCP g/Kg DM	912^a^	895^a^	894^ab^	867^b^	890^a^	5.12	0.0068	0.0096
DNDF, g/Kg DM	792	769	757	693	757	13.62	0.2380	0.2090
DS, g/100 g	99.3^a^	99.3^a^	99.2^ab^	99.1^b^	99.3^a^	0.343	0.0120	0.1375

Control = diet without additive; EY = enzyme + yeast; TE = tannin + enzyme; TY = tannin + yeast; TYE = tannin + yeast + enzyme; SEM = standard error of the mean; DMI = dry matter intake; BW = body weight; MBW = metabolic body weight; SI = starch intake; CPI = crude protein intake; NDFI = Neutral detergent fiber intake; DDM = digestibility dry matter; DCP = digestibility crude protein; DNDF = digestibility neutral detergent fiber; DS = digestibility starch. Means with different superscript lowercase letters (a, b) within a row differ significantly (*P* < 0.05). Control vs. Additive = contrast comparing the control treatment with the average of all additive-containing treatments (TE, EY, TY, and TYE)

Dry matter digestibility (DMD) was higher for diets containing additives than for the control diet, based on the planned contrast (control vs. additives; *P* = 0.0499), despite the absence of a significant overall treatment effect (*P* = 0.1346). In contrast, crude protein digestibility (CPD) was reduced (*P* = 0.0096). The combination of tannin and yeast also decreased CPD (*P* = 0.0068) and starch digestibility (DS; *P* = 0.012), although the reduction in DS was numerically small. No treatment effects were observed for water intake (WI), urinary volume (UV), fecal score (FS), urinary nitrogen excretion (UN), fecal nitrogen excretion (FN), or nitrogen balance (NB) (*P* > 0.05; Table 3). However, FN tended to be lower in additive-supplemented animals compared with the control treatment (*P* = 0.0939; Table 3).

**Table 3:** Water intake, urinary parameters and nitrogen balance of lambs fed diets with yeast, tannins, amylolytic enzymes, or without additive

Item	Control	EY	TE	TY	TYE	SEM	*p*-value	Control vs. Additive
WI, L/day*	4.34	3.93	3.15	3.97	4.26	0.7680	0.4952	0.6871
UV, L/day	1.38	2.39	2.28	2.04	2.02	0.2464	0.7255	0.2329
UN, %	6.04	7.94	7.46	6.03	5.93	0.3631	0.2584	0.4932
FN, %	3.02	3.04	3.15	3.23	3.19	0.0308	0.1887	0.0939
NB, %	0.042	0.038	0.045	0.039	0.044	0.0022	0.6180	0.9291
FS**	2.2	2.2	2.4	2.3	2.08	0.0677	0.7157	-

Control = diet without additive; EY = enzyme + yeast; TE = tannin + enzyme; TY = tannin + yeast; TYE = tannin + yeast + enzyme; SEM = standard error of the mean; *Analyze using log transformation and antilog; **non-parametric test. WI = water intake, UV = Urinary volume; UN = Urinary nitrogen; FN = Fecal nitrogen; NB = Nitrogen Balance = (nitrogen intake − (fecal nitrogen + urinary nitrogen)); FS = Fecal Score. Control vs. Additive = contrast comparing the control treatment with the average of all additive-containing treatments (TE, EY, TY, and TYE)

### Serum metabolites

The inclusion of feed additives did not affect serum concentrations of albumin, total protein, uric acid, creatinine, urea, AST, or energy-related metabolites (*P* > 0.05; Table 4). Plasma urea concentration showed a tendency to decrease in additive-supplemented animals compared with the control treatment.

**Table 4:** Serum metabolites of lambs fed diets with yeast, tannins, amylolytic enzymes, or without additive

Item	Control	EY	TE	TY	TYE	SEM	*p*-value	Control vs. Additive
Albumin, g/dL	0.506	0.716	0.528	0.567	0.65	0.0457	0.6036	0.3664
Total Protein, g/dL	6.52	6.46	6.48	6.5	6.58	0.0741	0.9359	0.8941
Urea, mg/dL	53.5	45.2	44.3	46	48.3	1.6985	0.2531	0.0627
Uric acid, mg/dL	0.373	0.355	0.352	0.389	0.395	0.0205	0.9556	0.9459
Creatinine, mg/dL	0.761	0.765	0.756	0.772	0.729	0.0221	0.9553	0.9188
AST, U/L	110	108	119	105	126	5.9893	0.7336	0.7627
GGT, U/L	25	29.4	28.5	29	27	0.6577	0.1338	0.0169
ALP, U/L	331	239	270	299	280	10.45	0.0746	0.0290
Fructosamine, mol/L	486	479	482	489	543	19.32	0.4545	0.6930
Triglycerides, mg/dL	17.8	16.7	16.8	15.5	19.5	0.6235	0.2823	0.7828
Cholesterol, mg/dL	54.3	54.3	58.4	51.3	55.8	2.6198	0.7580	0.8730
HDL, mg/dL	34.7	34.3	37.9	32.7	37	1.7047	0.4942	0.7519
LDL, mg/dL	20.3	19.5	21.2	18.9	19.7	0.8939	0.9143	0.8143

Control = diet without additive; EY = enzyme + yeast; TE = tannin + enzyme; TY = tannin + yeast; TYE = tannin + yeast + enzyme; SEM = standard error of the mean; AST = aspartate aminotransferase; GGT = gamma-glutamyl transferase; ALP = alkaline phosphatase; HDL = high-density lipoprotein; LDL = low-density lipoprotein; VLDL = very-low-density lipoprotein. Control vs. Additive = contrast comparing the control treatment with the average of all additive-containing treatments (TE, EY, TY, and TYE)

Although the overall treatment effect was not significant, the planned contrast (control vs. additives) indicated greater GGT activity (*P* = 0.0169) and lower ALP concentration (*P* = 0.0290) in animals fed diets containing additives than in those receiving the control diet. Despite these changes, all values remained within the physiological reference ranges reported for lambs.

## Discussion

### Feed intake, digestibility, and nitrogen balance

The inclusion of feed additives did not affect nutrient intake in lambs. The absence of treatment effects on nutrient intake may reflect the similar composition and physicochemical characteristics of the experimental diets, which likely contributed to maintaining comparable intake across treatments. The average DMI observed in this study was higher than that recommended by Pereira et al. (2024) for this animal category (0.866 kg day⁻¹). This greater intake may be attributed to the high proportion of concentrate in the diet, which presents higher degradation rates and smaller particle size, increasing solubility and passage rate. These conditions may accelerate ruminal fermentation and reduce retention time, thereby promoting higher voluntary intake (Macedo Júnior et al. 2007).

According to the planned contrast (control vs. additives), dry matter digestibility was higher in additive-supplemented diets. Although the underlying mechanisms were not directly assessed, this response may be associated with interactions among the additives that influenced nutrient digestion. Yeast supplementation may stabilize ruminal pH by scavenging oxygen and stimulating the growth of fibrolytic and lactate-utilizing bacteria, thereby improving fermentation efficiency (Bidarkar et al. 2014). In addition, amylolytic enzymes may enhance starch hydrolysis, increasing substrate availability for microbial fermentation and energy supply (López-Aguirre et al. 2016; Salem et al. 2011; Neiva et al. 2022). Tannins, in turn, may modulate ruminal fermentation by selectively inhibiting proteolytic microorganisms, potentially improving nitrogen utilization efficiency (Ramdani et al. 2023; Patra and Saxena 2009). The combined use of these additives may have contributed to the observed changes in nutrient digestibility; however, the underlying ruminal mechanisms were not evaluated in the present study.

On the other hand, the combination of tannin and yeast reduced crude protein and starch digestibility. This effect is likely related to the binding capacity of condensed tannins, which form complexes with proteins and carbohydrates, limiting microbial access and enzymatic degradation in the rumen (Thi Mui Nguyen et al. 2005). Although part of this protein may be digested post-ruminally, the reduced ruminal degradation may explain the lower apparent digestibility. Additionally, tannin–carbohydrate interactions may impair starch availability, which could explain the reduction in starch digestibility. However, the magnitude of the change in starch digestibility was small, suggesting limited biological relevance under the conditions of this study. Similar findings have been reported by de Jong et al. (2025) and Berça et al. (2023). In contrast, treatments containing enzymes may have partially mitigated this effect, which could be related to the expected action of amylolytic enzymes on starch degradation.

Water intake (WI) did not differ among treatments, possibly due to the similar dry matter content (77%) of the diets. The observed WI was approximately 7% higher than the initial expectation based on DMI. This result is consistent with NRC (2007), which indicates that water intake in sheep generally ranges from two to three times DMI. Urinary volume (UV) was 2.2 times higher than the expected value for this animal category, according to Dukes et al. (2006), which may be attributed to the increased WI.

Regarding urinary nitrogen (UN), no significant differences were observed among treatments. Fecal nitrogen (FN) tended to decrease in treatments containing feed additives compared with the control group. This response may suggest alterations in nitrogen excretion patterns; however, the underlying mechanisms were not directly evaluated. Therefore, the observed reduction in FN should be interpreted with caution. Finally, fecal scores remained within the normal range proposed by Dickson and Jolly (2011) (score ≈ 2), indicating no evidence of gastrointestinal disturbances even under high-concentrate diets containing feed additives.

### Serum metabolites

Plasma urea concentration tended to decrease in lambs receiving dietary additives (*P* = 0.0627), which may suggest improved utilization of dietary nitrogen. The observed changes in nitrogen-related parameters occurred concurrently with the increase in dry matter digestibility; however, the causal relationship between these responses could not be established under the conditions of the present study. Previous studies have reported that yeast, tannins, and exogenous enzymes may influence nitrogen metabolism in ruminants (Halfen et al. 2021; Besharati et al. 2022; Ferreira et al. 2025; Thompson et al. 2025). Nevertheless, these mechanisms were not directly evaluated in the present study; therefore, they should be interpreted as potential explanations rather than direct evidence for the responses observed.

In high-starch diets, the non-enzymatic glycation of proteins may be enhanced due to increased availability of post-ruminal glucose. Proteins that are normally less susceptible to glycation may therefore become modified, as described by Jagadeeshaprasad et al. (2018), resulting in reduced serum albumin concentrations and increased fructosamine levels (Lundholm et al. 2020). This response appeared to be independent of the feed additives, as it occurred across all experimental groups.

According to the planned contrast (control vs. additives), additive supplementation resulted in greater GGT activity (*P* = 0.0169) and lower ALP concentration (*P* = 0.0290) than the control diet. However, all values remained within the physiological reference ranges reported for lambs (Varanis et al. 2021), indicating no evidence of hepatic dysfunction and suggesting limited biological relevance. These findings are consistent with those of Ribeiro et al. (2025), Freitas et al. (2024), Siqueira et al. (2022), and Rodrigues et al. (2021), who also reported no major alterations in hepatic enzyme activity following additive supplementation.

Overall, the use of feed additives in combination with a high-quality, high-intake diet was associated with changes in selected metabolic and nitrogen-related parameters, without evidence of adverse effects on animal health, as biochemical parameters remained within the reference ranges established by Varanis et al. (2021). However, these findings should be interpreted within the context of the controlled experimental conditions and the 5 × 5 Latin square design. In addition, the mechanisms underlying the observed responses were not directly evaluated. Therefore, further studies are needed to confirm these findings and better elucidate the biological mechanisms involved.

The use of yeast, tannin, and amylolytic enzymes in high-concentrate diets for lambs was associated with increased dry matter digestibility without affecting nutrient intake, water intake, nitrogen balance, or indicators of animal health. The combination of tannin and yeast reduced crude protein and starch digestibility, whereas supplementation altered selected biochemical parameters while remaining within physiological reference ranges. These findings partially support the proposed hypothesis and suggest that the combined use of these additives may influence nutrient utilization and metabolic responses without compromising animal health. However, given the experimental conditions of the present study, further research is warranted to confirm these responses and clarify the underlying biological mechanisms.

## Data Availability

Data are available on request only.
